# Factors associated with loss of motivation and hesitation to work amongst frontline health care providers during the COVID-19 pandemic: A cross-sectional survey from a developing country

**DOI:** 10.1016/j.amsu.2022.104766

**Published:** 2022-09-30

**Authors:** Mehreen Malik, Syeda Tayyaba Rehan, Farheen Malik, Jawad Ahmed, Chaudhary Abdul Fatir, Hassan ul Hussain, Asiyah Aman, Muhammad Junaid Tahir

**Affiliations:** a Aga Khan University Hospital, 74800, Karachi, Pakistan; b Dow University of Health Sciences, 74200, Karachi, Pakistan; c Lahore General Hospital, Lahore, 54000, Pakistan

**Keywords:** SARS-CoV-2, Doctors, Mental health, Stress, Depression, Knowledge

## Abstract

**Background:**

The COVID-19 took over the world in 2020 and a lockdown has been imposed seeing its fast spread. Frontline health care workers (HCWs) were reported frequently with a lack of motivation, hesitancy and unwillingness to perform their duties during this pandemic. This cross-sectional survey aims to evaluate the factors associated with lack of motivation and increased hesitancy among the frontline HCWs to perform their duties during COVID-19 pandemic.

**Materials and methods:**

A total of 239 HCWs were included in this web-based cross-sectional study, who have worked during the COVID-19 pandemic. The anonymous online questionnaire was sent to all faculty, trainees and staff of Aga Khan University Hospital in Karachi, Pakistan. The survey was conducted from September 2020 to January 2021 during the COVID-19 pandemic. All data was exported into Statistical Package for Social Sciences Version 19 for multivariate analysis.

**Results:**

The risk of getting infected was strongly reported by 180 participants, and it was associated with higher hesitation to work (aOR = 6.09 [2.55–14.59]). Fifty-one participants felt that lack of knowledge about prevention and protection was associated with lower motivation to work (aOR = 0.66 [0.35–1.25]). Participants reported higher hesitation due to the burden of changed quality of work, physical exhaustion, mental exhaustion and altered sleep patterns. Sense of feeling protected by their hospitals was a motivating factor, and participants receiving adequate support reported higher motivation to work (aOR = 2.60 [1.32–5.14]).

**Conclusion:**

Fear of infection, increased working hours, and inadequate support of the workplace played a key role in escalating the hesitancy among HCWs to perform their duties. Lack of disease knowledge and paucity of personal protective equipment further lowered the motivation levels of HCWs to work effectively during the COVID-19 pandemic.

## Highlights

• The COVID-19 took over the world in 2020 and a lockdown has been imposed seeing its fast spread. Frontline health care workers (HCWs) were reported frequently with a lack of motivation, hesitancy and unwillingness to perform their duties during this pandemic.
• This cross-sectional survey aims to evaluate the factors associated with lack of motivation and increased hesitancy among the frontline HCWs to perform their duties during COVID-19 pandemic.
• Fear of infection, increased working hours, and inadequate support of the workplace played a key role in escalating the hesitancy among HCWs to perform their duties.

## 1 Background

Coronavirus disease 2019 (COVID-19), the disease caused by severe acute respiratory syndrome coronavirus 2 (SARS-CoV-2), has sparked a global public health crisis. It has, in turn, challenged and exhausted the health care system around the world. As per World Health Organization's (WHO) online statistics, on June 10, 2022, there have been more than 53 million confirmed cases, including 6 million plus deaths due to this pandemic [1]. It is spread from person to person through aerosols produced by coughing or sneezing, nasal discharge, saliva, urine, and stool, as well as close contact with an infected person [2]. Several vaccines have been introduced for the public. However, due to the lack of availability in many parts of the globe, myths and false information spreading amongst the society, the standard operating procedures (SOPs) and vaccination system are not enough to combat this pandemic.

Since the first case of COVID-19 reported in Pakistan on February 26, 2020, the total tally has amounted to 1.5 million as of July 10, 2022 [3]. According to a study, it was evident that Pakistan did not have adequate measures in place for the challenging pandemic since the healthcare infrastructure and facilities were not, and still are not, up to the required standards [4]. With the scarcity of resources and protective gear, both patients and physicians are at risk, mainly due to the high transmission and infectivity rates by physical contact and respiratory droplets [5]. In addition, owing to the sudden increase in the workload of treating COVID-diagnosed patients, inadequate training, and the increased risk of contracting the infection and passing it to their families, the willingness to work in healthcare settings has been affected [6].

There are also significant psychological effects on the frontline health care workers (HCWs) due to the pandemic, which has affected their morale and mental health, thereby impacting the efficiency of an already challenged health care system [7]. A cross-sectional study conducted in Pakistan showed that during the COVID-19 pandemic, levels of anxiety in physicians are directly affected by greater exhaustion, increased family strain, and reduced feelings of protection [8]. Another study showed a 43% prevalence of anxiety among physicians within a month of recognizing the first case of COVID-19 [9]. Research on the willingness to work amongst HCWs in Bangladesh showed that around 30% either directly declined to work during the pandemic or were uncertain [10]. In another study, around 21% of HCWs of selected hospitals of Southwest Ethiopia were unwilling to work [11]. In other past studies of HCWs’ willingness to work in a pandemic, like the H1N1 influenza pandemic, up to 20% showed hesitation to work in China [12], while in other countries like Nigeria and Hong Kong, the percentages were at 66 and 77 respectively [13,14]. According to the study by Tahir et al., HCWs experience a high level of stress, depression, and anxiety during these periods of physical and mental overload during the pandemic which have long-term psychological impacts on their work and personal lives [15].

Hence, it is necessary to assess and address the factors leading to loss of motivation and hesitation in frontline HCWs to work during the pandemic, for which, to the best of our knowledge, no research has been done in Pakistan. It is vital to tackle both factors affecting motivation and hesitation simultaneously. As shown in an article by Imai et al., enabling factors that only increase motivation may lead to exhaustion in the long term while dealing with increased hesitation will only reduce the barrier to work in high-risk situations [16]. Our study will provide a basis for concerned authorities to take the necessary steps to deal with mental health struggles and acute burnout and improve healthcare delivery to the public.

## 2 Methodology

A questionnaire based electronic survey was conducted among frontline HCWs working in Aga Khan University Hospital, Karachi, Pakistan from September 2020 to January 2021 during the COVID-19 pandemic. The work has been reported in line with STROCSS criteria [17].

The questionnaire was developed after extensive literature review and previously published articles [[18], [19], [20], [21], [22], [23]] were set as an example to design a Google® Form questionnaire consisting of 28 items. The first section included a brief explanation of the study and informed consent. The second section covered major aspects of demographic variables related to the age, gender, department and job designation. The third section explored their perceptions about the COVID-19 outbreak, along with their hesitancy to work as healthcare providers while caring for COVID-19 patients. Questions regarding their hesitancy to work in these trying times were also included. Likert 4-point scale [0 = never, 1 = rarely, 2 = sometimes, 3 = always] was used to respond to the 28 items on the list.

The anonymous online questionnaire was sent to all faculty, trainees and staff of Anaesthesia, Medicine, Critical care and Emergency Departments of Aga Khan University Hospital in Karachi, Pakistan. Email requests were sent to the official (internal) inbox after approval from their departmental heads. Mental health referral numbers including current AKU Psychiatric helpline, run by the Psychiatry department, was provided in the emails. A pilot study was conducted by selecting a small sample of participants (n = 20) and the data was analysed to ensure the validity and reliability of questionnaire before data collection (Cronbach's alpha = 0.5). The data of the pilot study was not used for the final analysis. After a thorough discussion, the questionnaire was checked by a psychiatrist for relevance and simplicity, and the authors finalized the questionnaire ensuring its consistency with the previously published literature. Then it was distributed to the participants for their response and all participants gave their informed consent to participate in this study.

The study was carried out in accordance with the Helsinki Declaration. It was an observational study that valued the anonymity and autonomy of the participants. Participants were allowed to withhold the completed form from the submission or leave the form without completing. It was ensured that the privacy of each participant was adequately protected. As it was an online questionnaire-based study, a waiver was requested and obtained from the ethical review committee (ERC) of The Aga Khan University (AKU), Karachi, Pakistan for this non-interventional survey administration. [ERC approval number: 2020-5189-11863].

All data was exported into Statistical Package for Social Sciences Version 19 (SPSS Inc., Chicago, IL) for multivariate analysis. Responses of the loss of motivation and hesitation to work were dichotomized according to scale score. Mean and standard deviation were calculated for the quantitative variables. Frequency and percentages were calculated for gender, job types, and working place. Chi-square test was used to observe the association between factors and loss of motivation and hesitation to work. Logistic regression analysis was performed to adjust factors associated with loss of motivation and hesitation to work. Crude and adjusted odd ratio with 95% confidence interval was estimated for each factor to observe the strength of association. A p-value of less than 0.05 was considered statistically significant.

## 3 Results

### 3.1 Basic demographics

A total of 239 participants were included in the analysis, amongst which 128 have worked in the COVID-19 intensive care unit (ICU), ward, or outpatient department (OPD) while 111 had been employed in emergency departments or operation theatre (OT). Around two-fifths of the participants (42.7%) were residents, medical officers, or interns, while the rest of our respondents constituted faculty members (24.7%), nurses (25.1%), and technicians (7.5%). The study predominantly comprised females (50.6%). Other basic demographics of our respondents have been highlighted in Table 1.

**Table 1:** Factors associated with hesitation and motivation to work by univariate analysis.

Variables	n	High Hesitation		High Motivation	
		n (%)	UOR [95%CI]	n (%)	UOR [95%CI]
**Age Groups**					
≤30	**99(41.4%)**	36(36.4%)	Ref	32(32.3%)	Ref
31–40	**84(35.1%)**	36(42.9%)	1.31[0.72–2.38]	24(28.6%)	0.84[0.44–0.16]
41–50	**40(16.7%)**	11(27.5%)	0.66[0.29–1.48]	9(22.5%)	0.61[0.25–1.43]
>50	**16(6.7%)**	4(25%)	0.58[0.17–1.94]	5(31.3%)	0.95[0.31–2.97]
**Gender**					
Male	**118(49.4%)**	41(34.7%)	Ref	36(30.5%)	Ref
Female	**121(50.6%)**	46(38%)	1.15[0.68–1.95]	34(28.1%)	0.89[0.51–1.55]
**Job Classification**					
Faculty	**59(24.7%)**	20(33.9%)	Ref	12(20.3%)	Ref
Resident/Medical officer/Intern	**102(42.7%)**	40(39.2%)	1.25[0.64–2.46]	36(35.3%)	2.14[1.01–4.54] *
Nurse	**60(25.1%)**	22(36.7%)	1.13[0.53–2.39]	15(25%)	1.31[0.55–3.09]
Technician	**18(7.5%)**	5(27.8%)	0.75[0.23–2.40]	7(38.9%)	2.49[0.79–7.79]
**Working**					
Emergency/OT	**111(46.4%)**	49(44.1%)	Ref	39(35.1%)	Ref
COVID-19 ICU/ward/OPD	**128(53.6%)**	38(29.7%)	0.53[0.31-.91] *	31(24.2%)	0.59[0.34–1.04]

*Statistically significant.

### 3.2 Sociodemographic factors associated with hesitation and motivation to work

Non-adjusted and adjusted ORs for factors associated with high hesitation and high motivation to work are shown in Table 1, Table 2, respectively.

**Table 2:** Multivariate analysis, factors associated with hesitation and motivation to work.

Variables	High Hesitation	High Motivation
	aOR [95%CI]	aOR [95%CI]
**Age Groups**		
≤30	Ref	Ref
31–40	1.29[672–2.48]	1.03[0.52–2.05]
41–50	0.52[0.18–1.45]	1.04[0.36–2.97]
>50	0.45[0.10–1.99]	1.74[0.40–7.45]
**Gender**		
Male	Ref	Ref
Female	1.12[0.68–2.18]	0.96[0.53–1.75]
**Job Classification**		
Faculty	Ref	Ref
Resident/Medical officer/Intern	0.79[0.21–1.99]	2.54[0.91–7.09]
Nurse	0.80[0.32–1.99]	1.62[0.57–4.53]
Technician	0.39[0.11–1.48]	2.32[0.64–8.41]
**Working**		
Emergency/OT	Ref	Ref
COVID-19 [ICU/ward/OPD]	0.44[0.25–0.78] *	0.63[0.34–1.14]

a OR = Adjusted Odd Ratio *Statistically Significant.

#### 3.2.1 Age groups and gender

Most of the participants from our study were ≤30 years of age. Amongst all age groups, except those >50 years of age, hesitation to work in the hospital was found to exceed the motivation factor. The respondents in the age group of 31–40 years were observed to have the highest level of hesitation (UOR = 1.31[0.72–2.38]; aOR = 1.29[672–2.48]), whereas high motivation was seen in >50 years age group compared to 31-40- and 40-50-years age groups (Table 1, Table 2).

The female participants in our study indicated a higher degree of hesitation than their male counterparts (Table 1, Table 2).

#### 3.2.2 Job classification and working

As highlighted in Table 2, the study group comprising of residents, medical officers, and interns was significantly associated with higher motivation levels (aOR 2.54[0.91–7.09]) as compared to the other subgroups.

The employment of study participants in the COVID-19 ward, ICU, or OPD was significantly associated with lower levels of motivation (aOR 0.63[0.34–1.14]) compared to Emergency/OT. The ORs for other factors and subgroups can be seen in Table 1, Table 2

### 3.3 Stress factors and the likelihood of reporting hesitation or motivation to work

**Fig. 1:**
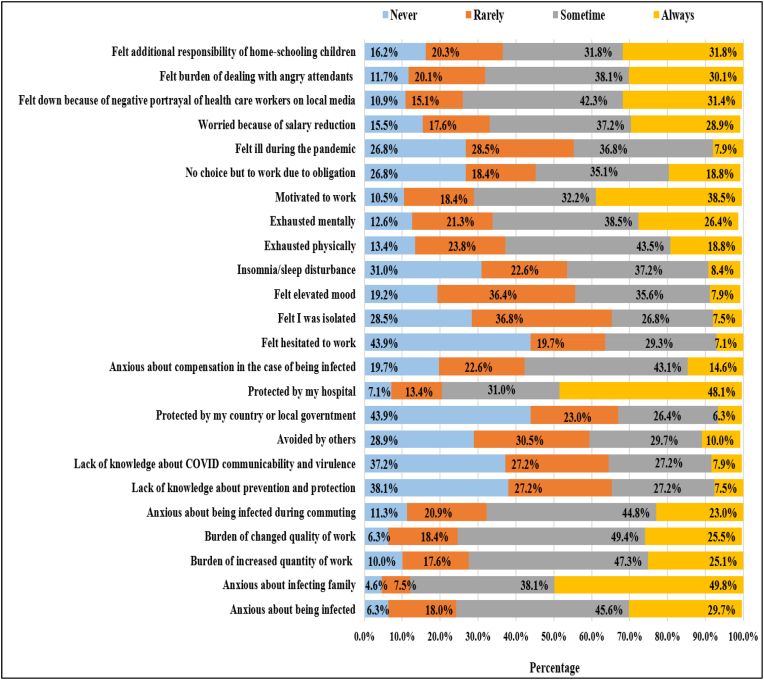
Response of participants.

About one-third (29.7%) of participants reported being always anxious about getting infected with COVID-19, while almost half (49.8%) participants are always in fear of infecting their family. About 39.7% of participants felt that they were avoided by others. About 19.2% of participants reported that they never felt elevated mood working amidst COVID-19, 26.4% of participants reported being mentally exhausted, and 18.8% were always physically exhausted. Over 40% of participants reported sleep disturbances/insomnia, and about two-thirds of participants felt worried because of salary reduction. Fig. 1 summarizes the response of participants to different stress factors.

Table 3 shows associations of different stress factors (e.g., risk of infection, knowledge, protection, isolation, etc.) and the likelihood of reporting hesitation and motivation to work.

**Table 3:** Associations of stress factors and likelihood of reporting hesitation and motivation to work.

	Weak	Strong	Among the strong		Hesitation to work		Motivation to work	

	n(%)	n(%)	High Hesitation	High Motivation	UOR [95%CI]	†aOR [95%CI]	UOR [95%CI]	†aOR [95%CI]
**Risk of infection**								
Anxious about being infected	58	180	80	51	5.82	6.09	0.88	0.93
	(24.4)	(75.6)	(44.4)	(28.3)	[2.51–13.54]	[2.55–14.59]	[0.46–1.67]	[0.47–1.84]
Anxious about infecting family	29(12.1)	210(87.9)	83(39.5)	55(26.2)	4.08 [1.37–12.16]	4.28 [1.37–13.34]	0.33 [0.15–0.73]	0.32 [0.14–0.77]
Anxious about being infected during commuting	77	162 (67.8)	77 (47.5)	46 (28.4)	6.06 [2.92–12.63]	6.15 [2.86–13.21]	0.87 [0.48–1.58]	0.84 [0.45–1.56]
	(32.2)							
**Knowledge and measurement**								
Lack of knowledge about prevention and protection	156	83	51	21	4.65	5.34	0.74	0.66
	(65.3)	(34.7)	(61.4)	(25.3)	[2.62–8.23]	[2.89–9.84]	[0.41–1.34]	[0.35–1.25]
Lack of knowledge about COVID communicability and virulence	154	84	50	22	4.65	5.09	0.78	0.74
	(64.7)	(35.3)	(59.5)	(26.2)	[2.62–8.23]	[2.77–9.35]	[0.43–1.42]	[0.40–1.36]
**Protection**								
Protected by my country or local government	78	160	48(	51	0.43	0.44	1.45	1.55
	(32.8)	(67.2)	30)	(31.9)	[0.24–0.75]	[0.25–0.78]	[0.78–2.68]	[0.82–2.93]
Protected by my hospital	189 (79.1)	49 (20.5)	13 (26.5)	22 (44.9)	0.56 [0.27–1.13]	0.58 [0.25–1.20]	2.45 [1.28–4.72]	2.60 [1.32–5.14]
Anxious about compensation in the case of being infected	101 (42.3)	138 (57.7)	74 (53.6)	38 (27.5)	7.82 [3.99–15.31]	8.31 [4.12–16.78]	0.82 [0.46–1.44]	0.76 [0.43–1.37]
**Condition**								
burden of increased quantity of work	66 (27.6)	173 (72.4)	82 (47.4)	53 (30.6)	10.99 [4.21–28.69]	13.54 [4.73–38.74]	1.27 [0.67–2.41]	1.23 [0.61–2.46]
Burden of changed quality of work	59 (24.7)	180 (75.3)	85 (47.2)	52 (28.9)	25.5 [6.04–107.63]	25.12 [5.82–108.27]	0.92 [0.48–1.75]	0.92 [0.46–1.82]
Exhausted physically	89 (37.2)	150 (62.8)	78 (52)	45 (30)	9.63[4.50–20.59]	10.63 [4.74–23.86]	1.09 [0.61–19.5]	1.04 [0.56–1.93]
Exhausted mentally	81 (33.9)	158 (66.1)	79 (50)	48 (30.4)	9.12 [4.13–20.18]	8.51 [3.72–19.41]	1.17 [0.65–2.12]	1.07 [0.56–2.05]
Insomnia/sleep disturbance	128 (53.6)	111 (46.4)	64 (57.7)	32 (28.8)	6.22 [3.45–11.19]	7.92 [4.10–15.32]	0.96 [0.55–1.67]	0.87 [0.48–1.58]
Felt elevated mood	104	133 (56.1)	31 (23.3)	44 (33.1)	0.26 [0.15–0.45]	0.25 [0.14–0.45]	1.56 [0.87–2.78]	1.56 [0.86–2.83]
	(43.9)							
**Isolation**								
Avoided by others	142	95	52	27	3.8	4.36	0.95	0.91
	(59.4)	(39.7)	(54.7)	(28.4)	[2.19–6.71]	[2.67–8.04]	[0.53–1.68]	[0.49–1.67]
Felt I was isolated	156	83	60	23	12.46	13.11	0.89	0.86
	(65.3)	(34.7)	(72.3)	(27.7)	[6.61–23.51]	[6.71–25.64]	[0.49–1.60]	[0.46–1.58]
**Others**								
No choice but to work due to obligation	108 (45.2)	129 (54)	72 (55.8)	43 (33.3)	7.83 [4.10–14.95]	8.34 [4.15–16.78]	1.58 [0.88–2.79]	1.50 [0.81–2.77]
Felt ill during the pandemic	132	107	52	28	2.62	2.62	0.75	0.63
	(55.2)	(44.8)	(48.6)	(26.2)	[1.52–4.50]	[1.48–4.63]	[0.43–1.34]	[0.35–1.15]
Worried because of salary reduction	79	158	68	48	2.56	2.44	1.13	1.21
	(33.1)	(66.7)	(43)	(30.4)	[1.38–4.73]	[1.26–4.71]	[0.62–2.05]	[0.64–2.31]
Felt down because of negative portrayal of health care workers on local media	62	176	73	46	2.43	2.52	0.56	0.54
	(26.1)	(73.9)	(41.5)	(26.1)	[1.25–4.73]	[1.26–5.03]	[0.31–1.03]	[0.28–1.01]
Felt burden of dealing with angry attendants	76	163	71	40	2.89	2.95	0.49	0.48
	(31.8)	(68.2)	(43.6)	(24.5)	[1.54–5.45]	[1.53–5.71]	[0.28–0.89]	[0.27–0.89]
**If you have a child or children (n = 148)**								
Felt additional responsibility of homes schooling children (n = 148)	54	94	40	18	1.93	2.25	0.44	0.38
	(36.5)	(63.5)	(42.6)	(19.1)	[0.93–3.96]	[0.99–5.12]	[0.20–0.93]	[0.16–0.90]

†Adjusted for age, sex, job, and working status UOR= Unadjusted Odds ratio; aOR = adjusted odds ratio.

The risk of getting infected was strongly reported by 180 participants, and it was associated with higher hesitation to work (aOR = 6.09 [2.55–14.59]). Fifty-one participants felt that lack of knowledge about prevention and protection was associated with lower motivation to work (aOR = 0.66 [0.35–1.25]). Sense of feeling protected by their hospitals was a motivating factor, and participants reported higher motivation to work (aOR = 2.60 [1.32–5.14]). Participants were more likely to be hesitant to work if they felt anxious about being compensated in case of infection (aOR = 8.31 [4.12–16.78]). Insomnia/sleep disturbances are likely to lead to lower motivation to work (aOR = 0.87 [0.48–1.58]). Similarly, participants reported higher hesitation due to the burden of changed quality of work, physical exhaustion, and mental exhaustion. Working in the COVID-19 environment made HCWs feel isolated, which led to lower motivation (aOR = 0.86 [0.46–1.58] and higher hesitation (aOR = 13.11 [6.71–25.64]) to work.

The negative portrayal of HCWs on social media negatively impacted participants' motivation (aOR = 0.54 [0.28–1.01]) and made them feel more hesitant (aOR = 2.52 [1.26–5.03]) to work. Furthermore, the burden of dealing with angry attendants also reduced participants' motivation to work (aOR = 0.48 [0.27–0.89]).

Of 239 participants, 148 (61.9%) reported having one or more children. The additional responsibility of home-schooling children due to COVID-19 made participants more hesitant towards their work (aOR = 2.25 [0.99–5.12]). Details of all other stress factors and their associations with hesitation and motivation are reported in Table 3.

## 4 Discussion

This cross-sectional survey aims to assess the factors associated with lack of motivation and hesitancy among the health care workers to perform their duties during the COVID-19 pandemic. We found that lack of motivation and hesitancy to work during the COVID-19 pandemic was highly linked with fear of getting infected with this deadly virus. Out of 239 participants, one third of doctors reported always feeling anxious about contracting the virus at the workplace which made them hesitant to work. 49.8% of the participants showed concerns that they might infect their families and felt hesitant to work because of it. A similar survey conducted among Pakistani doctors showed a significantly positive relation between fear of COVID-19 and workplace avoidant behaviour among HCWs (*r* = 0.64, *p* < 0.01) [24]. The survey conducted by Malik et al. indicated that among doctors, fear of COVID-19 predicted 29.6% in workplace avoidance behaviour [24]. The studies found that the most common reason for stress and anxiety among health care professionals treating COVID-19 patients was the fear that they might infect their family members (89.2%), followed by the fear of getting infected themselves (80.3%) [25]. A study conducted in six hospitals of Ethiopia showed that 90% of HCWs felt worried about their loved ones and around 89% felt worried about losing someone due to covid-19 [25].

It was observed that lack of motivation and hesitancy to work during the COVID-19 pandemic among HCWs was stemming from a lack of awareness or dearth of support and protection provided from the workplace [26]. In this present study we found that the lack of awareness among doctors regarding the precautions and protective measures against virus made the workers anxious about their safety during work hours. This anxiety further decreased their motivation levels to work. In this survey, 51 doctors reported their lack of knowledge regarding this deadly disease decreased their motivation levels to work. The inadequate amount of personal protective equipment (PPE) provided to doctors played a large role in lowering their morale to work effectively during the pandemic. The lack of PPE escalated the mental fear among doctors to contract the virus and further infect their families and thus resulted in a hesitancy to work in hospitals. 62.5% of doctors reported feeling unsafe and prone to the virus due to lack of personal protective equipment in a survey by Sandhesh et al. [27].

In our study, we discovered that support and protection from the hospitals is a major factor to enhance the motivation levels to work among the doctors. In another study, doctors who received insufficient facilities from the workplace during the pandemic were more likely to suffer from depression which could further decrease their morale to work properly [28].

Our survey reveals that doctors felt isolated and cut off from their routine communications during the pandemic and this led to compromised mental health and negative effects on their quality of work. In this survey, around 39.7% of doctors reported that they were avoided by others. The lack of communication with doctors during this period could be to avoid the chances of infection by the local population but the contrary results have been seen in other studies. In a survey by Alnazly et al., the participants perceived high levels of support from all associated sources [28]. Doctors showed that they felt more supported and understood by their families during the COVID-19 pandemic. Health care workers from a cross-sectional study in Jordan indicated high recognition and support from friends during the time of the pandemic [28].

Due to the increased working hours or changes of work routine 26.4% of participants reported being mentally exhausted and 18.8% with physical exhaustion. Compromised mental and physical health further made the workers less motivated and hesitant to perform their duties. Similarly, another study reported HCWs suffered from mental disorders and physical fatigue during pandemic more than before [28]. Based on the external data, approximately 35% of the participants had extremely severe depression, over 40% had moderate to severe depression, and approximately 20% had normal to mild depression during the pandemic phase [28]. For anxiety, approximately 60% of the participants reported extremely severe anxiety. However, approximately 35% were severely distressed [28].

We observed that, increased burden of work and changed routine presented with insomnia and sleep disturbances in 40% of HCWs during the pandemic. Participants reported higher hesitation due to the burden of changed quality of work, physical exhaustion, and mental exhaustion. Participants were more likely to be hesitant to work if they felt anxious about being compensated in case of infection (aOR = 8.31 [4.12–16.78]). During the COVID-19 pandemic, nearly all the frontline HCWs surveyed on social media reported poor sleep, over one-third reported insomnia and over half reported burnout [29]. Insomnia and altered sleep patterns put forward the loss of motivation among doctors to perform well during hospital work hours.

During the pandemic due to the closure of activities and businesses, HCWs felt more concerned regarding their financial status and around two third of participants felt worried because of the salary reductions. The increased burden of work and reduction in salary packages, seen to be linked with lower motivations among HCWs.

We explored that the negative portrayal of health care professionals during pandemics on media networks pushed the doctors towards the mentally compromised state and hesitated them to work properly. The lack of awareness and increasing anxiety among the population due to shortage of space in hospitals made them blame the health care workers and resulted in hesitant behaviour of doctors during work.

In addition, 16.9% participants in this present study have one or more children which tend to increase their home responsibilities and make them more hesitant to work. This factor could be linked with concerns of parents towards the wellbeing of their children while their work hours escalated during the pandemic. It was also found that female doctors felt more concerned about their child home schooling during their long hours of work shifts. A study reported, married participants returned significantly higher scores for fear, depression, anxiety, and stress, respectively, when compared to single participants during the pandemic [28]. All these factors were likely to increase the hesitancy among doctors to perform well during work. (p = 0.015, p = 0.004, p = 0.019, and p = 0.012, respectively) [28].

This study has some limitations. First, as this was an online cross-sectional survey which involves HCWs of single institute, authors were not able to maintain a casual inference and a selection bias may be present. Second, there was not any assessment for pre-existing psychiatric complications in participants. Third, our sample size was small. Last, the mental assessment of HCWs was based on a self-designed (psychiatrist approved) questionnaire and not on neurological imaging and other psychiatric protocol was not fully followed to establish a concrete diagnosis of mental illness.

## 5 Conclusion

A detailed survey of HCWs during the COVID-19 pandemic drove us to the fact that fear of infection, increased working hours, and inadequate support of the workplace played a key role in escalating the hesitancy among HCWs to perform their duties. Lack of disease knowledge and paucity of personal protective equipment further lowered the motivation levels of doctors to work effectively during the COVID-19 pandemic.

## Ethical approval

Ethical approval was obtained from Ethical review committee (ERC) of The Aga Khan University (AKU), Karachi, Pakistan [ERC approval number: 2020-5189-11863].

## Sources of funding for your research

No external funding was received.

## Author contribution

Conceptualization: MM,STR, FM Data curation, formal analysis, investigation: FM, MM, JA, STR Methodology: FM, JA, CAF, MJT Validation: AA, CAF, MJT, MZS Visualization: AA, CAF, MZS, MJT Writing- original draft: FM, STR, MM, HH, JA, CAF Writing-review and editing: MJT, CAF, MZS, AA All authors have approved the final version of the manuscript.

## Registration of research studies

1. Name of the registry: N/A
2Unique Identifying number or registration ID: N/A
3. Hyperlink to your specific registration (must be publicly accessible and will be checked): N/A

## Guarantor

Muhammad Junaid Tahir.

## Consent

Informed consent was obtained from each participant in the first section of the survey form.

## Funding

None to declare.

## Provenance and peer review

Not commissioned, externally peer-reviewed.

## Declaration of competing interest

All authors declare no conflict of interests.
